# Epidemiological characteristics and comparative outcome of blast versus gunshot injuries of the extremities in Somalia

**DOI:** 10.1186/s13018-023-03527-9

**Published:** 2023-01-16

**Authors:** Abdullahi Yusuf Mohamed, Hassan Salad Ibrahim, Hüseyin Taşkoparan, Yasin Barkhad Ibrahim

**Affiliations:** Department of Orthopedics and Traumatology, Mogadishu Somali Turkish Training and Research Hospital, Nasib-Bundo Street, Shibis District, Mogadishu, Somalia

**Keywords:** Blast, Gunshot, Trauma, Extremity, Somalia

## Abstract

**Background:**

War conflicts and terror-related injuries constitute a significant public health problem in Somalia. We aim to characterize and compare the injury characteristics of gunshot and blast injuries of the extremities.

**Methods:**

The data of 333 patients with gunshot and blast injuries of the extremities over three years were retrospectively reviewed. The demographics, injury characteristics, and outcomes were analyzed.

**Results:**

Most of the patients had injuries due to gunshot casualties compared with blast victims (*n* = 222, 66.7% vs. *n* = 111, 33.3%). Patients with gunshot wounds (GSW) had a more significant proportion of males than those with blast wounds (BW) (95.5% vs. 85.6%, *P* < 0.001). There were more open extremity fractures in GSW casualties (96.4% vs. 81.1%). The BW victims had significantly higher associated injuries (52.3% vs. 18.5%, *P* < 0.001). The BW group had a higher injury severity score (ISS ≥ 16 in 55%, *P* < 0.001). The need for an intensive care unit (ICU) admission was significantly higher in the BW patients (18% vs. 6.3%, *P* < 0.001); as well as the length of hospital stay (LOS) was higher in the BW group compared with the GW patients (> 2-week hospital stay in 31% vs. 19%, *P* < 0.04). About a 2.7% mortality rate was observed in BW (*P* < 0.014).

**Conclusion:**

Gunshot and explosion injuries comprise the majority of war and terror-related trauma of the extremities. These injury mechanisms differ in the body regions involved, the severity of the injury, duration of hospital stay, need for ICU admission, and mortality. Assessment and management of such devastating casualties require a complex and multidisciplinary approach.

## Introduction

Trauma contributes to a significant burden of health care in developing and resource-limited countries, representing more than 90% of worldwide injury-related deaths [1], in which civilians are the most vulnerable to injuries on the modern battlefield [2]. Previous studies show that extremity injuries are most of the battlefield wounds encountered by the US armed forces in the Iraq and Afghanistan conflicts of the last decades [3–5].

Blasts and fires of different causes account for more than seventy percent of disasters, causing > 20% of deaths at a scene [6]. Whether accidental or intentional, blasts are incidents that can inflict severe injuries on many people, resulting in increased levels of morbidity and mortality. In the USA, more than 1200 deliberate bombings have been reported annually since 1991 [7]. Since the second world war, approximately 70% of wounds in conflicts are due to explosions. However, during the early phases of conflicts, gunshot injuries are more frequently met [8]. Arab springs have created an increasing and continuous number of war-related injuries. Over the past decade, Iraq and Syria encountered an increasing number of blast injuries affecting different body parts, including the extremities, where the resources to treat such injuries are limited [9]. The widespread applications of Improvised Explosive Devices (IEDs) gave rise to new injury patterns with devastating wounds, including high-energy extremity trauma [10]. Gunshot injuries are a significant cause of morbidity, which results in negative socioeconomic issues [11]. Gunshot injuries are three times more frequent in developing and low-resource-income countries compared to the United States and Europe, with its epicenter of gunshot violence burden located in Central and South America and South Africa [12]. Somalia, where its health infrastructure has collapsed, experienced longstanding war conflicts and terror-related injuries that claimed the lives of thousands of people and caused disabilities [13]. The extremities are the most commonly injured areas in battlefield conflicts [1, 2, 8], and information regarding injury characteristics and comparative outcomes of gunshot versus blast injuries of the extremities is limited [4, 5, 8].

This study aims to describe and compare the injury characteristics of the gunshot wounds (GSW) and blast injuries of the extremities in Somalia.

## Methods

In this study, the data of the patients with GSW and blast injuries of the extremities who were admitted to the orthopedic department of Mogadishu Somali Turkish Training and Research Hospital (MSTTRH) for three years (July 2018–June 2021) was analyzed retrospectively. The data were collected using the Hospital Information Management System (HIMS) software, and the patients with insufficient information were excluded from the analysis. A net of 333 casualties was included in the study. The study was approved by the institutional ethics committee, and the study was conducted in accordance.

The HIMS database was abstracted to obtain the following information for each patient: age, sex, mechanism of injury, the extremity of injury, associated injury, injury severity, open fracture characteristics, vascular injury, nerve injury, length of hospital stay (LOS), intensive care unit (ICU) admission, surgical procedure, early complication, and mortality. The international classification of diseases, ninth revision, (ICD-9) served as the basis for medical diagnosis classification. All patients were also examined using the injury severity score (ISS) based on the highest three most injured body regions of the abbreviated injury scale (AIS). The AIS assigns for each region points from 1 to 6. Injuries with AIS scores of 1 or 2 are minor and rarely cause death. Injuries with AIS scores of 3, 4, and 5 have increased severity, respectively. Those injuries with AIS scores of 6 are considered incompatible with life. ISS refers to a general score of 0–75, which indicates the severity of the injury and has been evaluated as follows: minor injury if 1 to 8, moderate injury if 9 to 15, severe injury if 16 to 24, and critical if 25 to 75 [14].

Data retrieved during the retrospective evaluation were analyzed using descriptive and inferential tests. The frequencies and proportions were presented as point estimates using Pearson’s chi-square test for categorical variables. Regarding the quantitative variables, mean ± standard deviation (mean ± SD) was expressed wherever necessary. Binary logistic regression model was used to assess the association between independent and dependent variables. The odds ratios (ORs) and their 95% confidence intervals (CIs) were computed. A *P*-value below 0.05 was considered statistically significant. All statistical analyses were performed using the Statistical Package for the Social Sciences (SPSS) v.26.0 software.

## Results

During the study, 333 patients were recorded with extremity traumas related to gunshot injuries and blast wounds (BW). Two third of the cases had injuries due to gunshot casualties. Most affected patients were males. Compared with the BW group, patients with GW had a more significant proportion of males (*P*-value < 0.001, 95% CI 1.097–8.019, OR 2.966), as demonstrated in Table 1. There was no statistically significant difference in age between the two groups.

**Table 1:** Patient demographics in GSW group versus BW group

Variable	No. of patients/%	Mechanism of injury		*P*-value	95% CI	OR
		GSW (*n* = 222)	BW (*n* = 111)			
*Age in years*				0.08	0.892–1.693	1.229
0–14	8 (2.4%)	4 (1.8%)	4 (3.6%)			
15–29	189 (56.8%)	130 (58.6%)	59 (53.2%)			
30–44	100 (30%)	68 (30.6%)	32 (28.8%)			
45–59	27 (8.1%)	18 (8.1%)	9 (8.1%)			
60–74	8 (2.4%)	2 (0.9%)	6 (5.4%)			
> 74	1 (0.3%)	0 (0%)	1 (0.9%)			
*Gender*				0.001	1.097–8.019	2.966
Male	307 (92.2%)	212 (95.5%)	95 (85.6)			
Female	26 (7.8%)	10 (4.5%)	16 (14.4%)			

Combined upper and lower extremity injuries were quite common in BW patients, whereas cases with gunshot injuries scored less (*P*-value < 0.002, 95% CI 0.863–5.535, OR 2.186). In both groups, the lower extremity was the most affected limb. There was no statistically significant difference regarding the anatomic region of injury among the victims of both GSW and BW. The pattern of associated injuries was significantly different between groups. The BW victims had more associated injuries compared with the GW group (*P*-value < 0.001, 95% CI 1.183–1.669, OR 1.405) and included more head and neck, chest, burn, and multisite injuries (Table 2).

**Table 2:** Distribution of musculoskeletal injuries and associated injuries

Variable	No. of patients/%	Mechanism of injury		*P*-value	95% CI	OR
		GSW (*n* = 222)	BW (*n* = 111)			
*Injured extremity*				0.002	0.863–5.535	2.186
Upper limb	141 (42.3%)	101 (45.5%)	40 (36.0%)			
Lower limb	184 (55.3%)	120 (54.1%)	64 (57.7%)			
Both limps	8 (2.4%)	1 (0.5%)	7 (6.3%)			
*Anatomic region*				0.02	0.955–1.468	1.184
Shoulder	10 (3.0%)	8 (3.6%)	2 (1.8%)			
Humerus	70 (21.0%)	54 (24.3%)	16 (14.4%)			
Forearm	41 (12.3%)	23 (10.4%)	18 (16.2%)			
Hand	27 (8.1%)	19 (8.6)	8 (7.2%)			
Pelvis	4 (1.2%)	2 (0.9%)	2 (1.8%)			
Femur	90 (27.0%)	63 (28.4%)	27 (24.3%)			
Patella	4 (1.2%)	1 (0.6%)	3 (2.7%)			
Tibia	77 (23.1%)	47 (21.2)	30 (27.0%)			
Foot	10 (3.0%)	5 (2.3%)	5 (4.5%)			
*Associated injury*	99 (29.7%)	41 (18.5%)	58 (52.3%)	0.001	1.183–1.669	1.405
No	234 (70.3%)	181 (81.5%)	53 (47.7%)			
Head and neck	15 (4.5%)	8 (3.6%)	7 (6.3%)			
Chest	12 (3.6%)	6 (2.7%)	6 (5.4%)			
Abdomen	5 (1.5%)	5 (2.3%)	0 (0%)			
Extremity	33 (9.9%)	15 (6.8%)	18 (16.2%)			
Multiple body regions	29 (8.7%)	5 (2.3%)	24 (21.6%)			
Burns	3 (0.9%)	0 (0%)	3 (2.7%)			
Spine	2 (0.6%)	2 (0.9%)	0 (0%)			

Regarding the musculoskeletal injuries themselves, there was a higher proportion of open extremity fractures in GW casualties, whereas the incidence rate of closed extremity fractures was significantly lower than the BW victims (*P*-value < 0.001, 95% CI 0.198–0.693, OR 0.351). High energy trauma was extensive in BW compared with GW (*P*-value < 0.02, 95% CI 0.837–3.169, OR 1.629). Soft tissue damage > 10 cm tended to be more common in BW casualties, whereas gunshot injury victims had commonly soft tissue wounds of 1-10 cm (*P*-value < 0.001, 95% CI 0.467–2.155, OR 1.003). Regarding wound contamination, blast injuries were related to extensive contamination, while gunshot injuries scored less extensive contamination (*P*-value < 0.001, 95% CI 0.807–4.329, OR 1.869). The incidence of vascular and nerve injuries was similar among the two groups (Table 3).

**Table 3:** Injury characteristics related to GSW versus BW

Variable	No. of patients/%	Mechanism of injury		*P*-value	95% CI	OR
		GSW (*n* = 222)	BW (*n* = 111)			
*Fracture*				0.001	0.198–0.693	0.351
No fracture	26 (7.8%)	8 (3.6%)	18 (16.2%)			
Closed	3 (0.9%)	0 (0%)	3 (2.7%)			
Open	304 (91.3%)	214 (96.4%)	90 (81.1%)			
*Energy*				0.02	0.837–3.169	1.629
Low	13 (3.9%)	11 (5%)	2 (1.8%)			
Moderate	155 (46.5%)	112 (50.4%)	43 (38.7%)			
High	165 (49.5%)	99 (44.6%)	66 (59.5%)			
*Wound size*				0.001	0.467–2.155	1.003
No wound	3 (0.9%)	0 (0%)	3 (2.7%)			
< 1 cm	41 (12.3%)	34 (15.3%)	7 (6.3%)			
1-10 cm	206 (61.9%)	147 (66.2%)	59 (53.2%)			
> 10 cm	83 (24.9%)	41 (18.5%)	42 (37.8%)			
*Contamination*				0.001	0.807–4.329	1.869
Clean	34 (10.2%)	28 (12.6%)	6 (5.4%)			
Moderate	203 (61%)	146 (65.8%)	57 (51.4%)			
Extensive	96 (28.8%)	48 (21.6%)	48 (43.2%)			
*Vascular*				0.8	0.243–2.649	0.803
No	257 (77.2%)	172 (77.5%)	85 (76.6%)			
Yes	76 (22.8%)	50 (22.5%)	26 (23.4%)			
*Nerve*				0.7	0.644–4.195	1.644
No	253 (76%)	170 (76.6%)	83 (74.8%)			
Yes	80 (24%)	52 (23.4%)	28 (25.2%)			

In comparing the severity of the injuries demonstrated in Table 4, the BW group had a higher proportion of casualties with an ISS of 16 or higher, whereas the GW group had more casualties with an ISS lower than 15 (*P*-value < 0.001, 95% CI 0.782–2.068, OR 1.272). The need for an intensive care unit admission was significantly higher in the BW patients (*P*-value < 0.001, 95% CI 0.281–2.246, OR 0.795), as well as the length of hospital stay, was higher in the BW group compared with the GW patients (*P*-value < 0.04, 95% CI 0.665–1.476, OR 0.991). There was a higher proportion of mortality in the BW group compared with the GW group (*P*-value < 0.01).

**Table 4:** ISS, ICU admission, LOS, and mortality of GSW versus BW

Variable	No. of patients/%	Mechanism of injury		*P*-value	95% CI	OR
		GSW (*n* = 222)	BW (*n* = 111)			
*ISS*				0.001	0.782–2.068	1.272
1–8	57 (17.1%)	37 (16.7%)	20 (18%)			
9–15	131 (39.3%)	101 (45.5%)	30 (27%)			
16–24	113 (33.9%)	74 (33.3%)	39 (35.1%)			
> 24	32 (9.6%)	10 (4.5%)	22 (19.8%)			
*ICU*				0.001	0.281–2.246	0.795
Yes	34 (10.2%)	14 (6.3%)	20 (18%)			
No	299 (89.8%)	208 (93.7%)	91 (82%)			
Mean ± SD		2 ± 2	4 ± 3			
*LOS* (*days*)				0.04	0.665–1.476	0.991
0–7	163 (49%)	111 (50%)	52 (46.8%)			
8–14	93 (27.9%)	68 (30.6%)	25 (22.5%)			
> 14	77 (23.1%)	43 (19.4%)	34 (30.6%)			
Mean ± SD		11 ± 10	13 ± 16			
*Mortality*				0.01	0.000	0.000
Yes	3 (0.9%)	0 (0%)	3 (2.7%)			
No	330 (99.1%)	222 (100%)	108 (97.3%)			

The proportion of definitive procedures was higher in the GW group, whereas the proportion of reoperations or secondary procedures, soft tissue reconstruction, and primary amputation was significantly higher in the BW group due to clinical conditions (*P*-value < 0.001, 95% CI 0.698–1.253, OR 0.935). The incidence of compartment syndrome, infection, and secondary amputations was similar among the two groups (Table 5).

**Table 5:** Operative management and complication

Variable	No. of patients/%	Mechanism of injury		*P*-value	95% CI	OR
		GSW (*n* = 222)	BW (*n* = 111)			
*Operation*				0.001	0.698–1.253	0.935
Debridement with direct closure or definitive fixation	156 (46.8%)	111 (50%)	45 (40.5%)			
Debridement with external fixation	86 (25.8%)	59 (26.6%)	27 (24.3%)			
Reoperations or secondary reconstruction	26 (7.8%)	10 (4.5%)	16 (14.4%)			
Primary amputations	35 (10.5%)	17 (7.7%)	18 (16.2%)			
Soft tissue reconstruction with fixation	2 (0.6%)	0 (0%)	2 (1.8%)			
Revascularization with fixation	28 (8.4%)	25 (11.3%)	3 (2.7%)			
*Infection*				0.09	0.449–2.040	0.957
Yes	72 (21.6%)	42 (18.9%)	30 (27%)			
No	261 (78.4%)	180 (81.1%)	81 (73%)			
*Secondary amputation*				1.0	0.117–3.062	0.597
Yes	12 (3.6%)	8 (3.6%)	4 (3.6%)			
No	321 (96.4%)	214 (96.4%)	107 (96.4%)			
*Compartment*				0.08	0.451–41.857	4.347
Yes	11 (3.3%)	10 (4.5%)	1 (0.9%)			
No	322 (96.7%)	212 (95.5%)	110 (99.1%)			

## Discussion

This study portrays the patients with extremity injuries who were hospitalized due to GSW and blast casualties in Somalia between July 2018 and June 2021. Epidemiological characteristics and comparative outcomes of the GSW versus BW casualties to the extremities were explored. The literature has reported that terror-related explosion injuries can result in multiple body injuries for four mechanisms; primary blast injury is mainly associated with damage to the air or fluid-filled organs such as the lungs and viscera. Penetrating objects from explosive devices cause secondary blast injuries, and these injuries are the most common cause of extremity injuries in our study. The fragments related to the secondary blast effects tend to be multiple and have higher energy depending on the distance of the victims to the area of detonation, such as open or closed space. The tertiary blast effects due to falling objects or collapsed structures cause blunt trauma, resulting in closed fractures in extremities. The quaternary blast injury causes burn and inhalational injuries [14–17]. Blast casualties in Somalia disclosed a previously unknown form of injury produced by new types of shrapnels, such as explosives or other sharp metal objects from vehicles in which explosives were planted. These sharp objects result in injuries that are difficult to be identified at the first trauma view prompting close observation and diagnostic screening. Total body fluoroscopy should be obtained from critical patients who need immediate operation theater transfer to detect metal foreign bodies that may complicate their course of treatment, whereas stable patients should have a total body computed tomography scan. On the other hand, gunshot injuries have distinctive ballistic properties resulting from different weapon systems and ammunition [18]. High-velocity gunshots can withhold half of their kinetic energy at a distance of 275 m from the muzzle. In contrast, the fragments from the blast generally have higher energy at the explosion point, but their energy reduces as they move away from the point of detonation [15]. Blast victims generally occur as mass casualties and arrive at the hospital as groups, while gunshot victims occur over a broad period and come to the hospital as sporadic. This difference has an essential association with hospital organizations and may affect patient care. In a mass casualty scenario, “minimally acceptable care” is provided. Minimally acceptable care focuses efforts on a maximal number of salvageable victims [17].

GSW was the predominant casualty, and in both groups, the demographics had polarized toward injured males (95.5%) as contrary to females. This figure is consistent with previous studies published in the literature [19–21]. The higher rate of male predominance may be due to male exposure to conflicts. Blast casualties most often involved multiple extremity injuries, whereas GSW was frequently involved in isolated specific body regions in our study. BW casualties have more associated injuries, including head and neck, thorax, multiple body regions, and burns, similar to a study reported previously in Israel [22]. A retrospective study examining the battlefield extremity injuries in Operation Iraqi Freedom reported that a higher percentage of all combatants suffered upper versus lower extremity injuries (47.3% vs. 43.2%). Explosions more often injured patients with concurrent upper and lower extremity wounds, and they sustained higher proportions of head/neck (50.3% vs. 33.1%), abdominal (13.8% vs. 5.6%), pelvis/urogenital (19.3% vs. 5.6%), and back/buttock injury (7.7% vs. 3.1%), each with statistical significance of *P* < 0.05. Similar to these reports our findings report that the combined upper and lower extremity injuries were higher in the BW group compared to the GW group but in contrary to these reports the distribution of the lower extremity blast injuries in our study were higher than the upper extremity injuries [4]. Similar to our results, a one-year retrospective study in Somalia shows that 91.2% of the fractures were open, while 8.8% were closed due to blast exposure. 40.4% of the open fractures were located in the upper extremities, and 59.6% were in the lower extremities and pelvis [14]. Multiple body region injuries are a significant cause of increased overall injury severity. Similar to previous studies, blast injuries were characterized by significantly higher rates of ISS, longer hospital stays, and more often the need for an intensive care unit. Our report indicates that the explosion victims have higher in-hospital mortality [15, 17, 22].

The limitation of this study includes a lack of functional assessment of patients according to the mechanism of injury and anatomical area. Only the patients admitted to the orthopedics and trauma department were analyzed in this study which excluded the patients with minor injuries who were discharged from the emergency department, as well as pre-hospital deaths and dead-on-arrival patients in which some salvageable victims may be missed. Another significant limitation of this study is that the patients could not be followed up after discharge from the hospital due to their low socio-economic level and lack of health insurance. Despite these limitations, our study presents a comprehensive work describing blast and gunshot-related extremity injuries in Somalia.

## Conclusion

Gunshot and explosion injuries comprise the majority of war and terror-related trauma in Somalia, which has been hosting conflicts for more than 30 years. Assessment and management of such devastating casualties require a complex and multidisciplinary approach. These findings have implications for the readiness of hospital resources and training to treat patients following terror attacks in any region.

Since our study is performed in a single center, studies relating to the prevalence of national trauma and tailored management of terror-related injuries are required to enhance the health staff's expertise and increase the patient's quality of care. As well as the implementation of a national trauma registry and improvements of decentralized trauma centers throughout the country is necessary to tackle terror-related casualties.
